# Effects of a brief online self-compassion training on perfectionism, self-criticism, and social anxiety: A randomized controlled trial

**DOI:** 10.1016/j.invent.2025.100870

**Published:** 2025-09-13

**Authors:** Kira S.A. Borgdorf, Corina Aguilar-Raab, Daniel V. Holt

**Affiliations:** a Psychological Institute, Heidelberg University, Hauptstraße 47-51, 69117, Heidelberg, Germany; b Department of Clinical Psychology, Interaction- and Psychotherapy Research, Institute for Compassionate Awareness and Interdependence Research and Practice IN-CARE, University of Mannheim, Willy-Brandt-Platz 1, 68161, Mannheim, Germany

**Keywords:** Self-compassion, Psychological health, Self-criticism, Perfectionism, Social anxiety, Randomized controlled trial

## Abstract

This randomized controlled trial evaluates the effects of a brief online self-compassion training (SCT) on self-compassion, self-criticism, perfectionism, social anxiety, and psychological health in comparison to a generic stress-reduction training (SRT). Both training courses consisted of six brief, format-matched, unsupervised, online sessions with various exercises, and took place in a self-paced manner over 2 to 4 weeks. We collected self-report data on self-compassion, self-criticism, perfectionism, social anxiety, and psychological health. Participants were 200 healthy adults (85.5 % female, *M*_age_ = 30 years), randomly allocated to the SCT or the SRT. In pre-post comparison, effect sizes for the SCT were moderate for self-compassion (*dz* = 0.49, 95 % CI [0.26, 0.72]), self-criticism (*dz* = −0.50, 95 % CI [−0.72, −0.28]), and perfectionism (*dz* = −0.41, 95 % CI [−0.62, −0.20]), but close to zero for social anxiety (*dz* = −0.01, 95 % CI [−0.21, 0.18]). Only small differences emerged between the conditions immediately after the training, except for self-compassion (*d* = 0.49, 95 % CI [0.02, 0.58]). At 4 weeks follow-up the effects of both trainings on the target variables, including self-compassion, were very similar. However, intervention-specific effects were pronounced and enduring for participants with high initial levels of self-criticism. The results indicate that both training courses yielded similar psychological effect patterns. Effects of the SCT were not specific to self-compassion and conceptually opposite variables like perfectionism or self-criticism. These findings highlight the importance of understanding core mechanisms of self-compassion interventions and identifying appropriate target groups in future research.

## Highlights

• The study examines a 4-week, unsupervised, online self-compassion training.
• The training led to increases in self-compassion and psychological health.
• The training led to decreases in perfectionism and self-criticism.
• The training was not superior to a matched active stress-reduction control training.

## 1 Introduction

Health is understood not only as the absence of disease, but the presence of complete physical, mental, and social well-being (World Health Organization [WHO], 1946). Yet, mental health research has shifted only recently from problem-oriented, curative approaches to mental health preservation and promotion (van Agteren et al., 2021). This randomized control trial examines to what extent a 4-week online Self-Compassion Training (SCT) fosters selective improvements in self-compassion, self-criticism, perfectionism, and social anxiety compared to a generic Stress-Reduction Training (SRT). The study thereby provides an investigation of transdiagnostic indicators of mental health and offers insights for the design of online mental health promotion trainings.

Self-compassion is defined as mindfully directing kindness and understanding towards oneself in difficult times and the ability to put perceived shortcomings into greater perspective (Neff, 2003, Neff, 2023). Contrarily, self-criticism entails a judgmental and critical attitude towards oneself with an emphasis on disapproving of perceived shortcomings and failings (e.g., Löw et al., 2020). Self-criticism is especially prevalent in perfectionism and social anxiety which are characterized by a pursuit of very high performance standards, an overly critical and harsh evaluation of own behaviour and performance, as well as a strong fear of failing (Schlenker and Leary, 1982; Stoeber, 2018). Both self-compassion and self-criticism have high transdiagnostic value, that is, both play a central role in multiple psychological disorders (Finlay-Jones, 2023; Löw et al., 2020): Self-compassion is considered a preventive factor (Han and Kim, 2023), and self-criticism a risk factor for several psychological problems and disorders, such as social anxiety, depression, and eating disorders (Löw et al., 2020).

People who report high self-compassion generally score highly on a range of desirable psychological variables such as happiness, optimism, well-being, life satisfaction, adaptive coping strategies, psychological health, and social connectedness (e.g., Asselmann et al., 2024; Ewert et al., 2021; Neff, 2023; Zessin et al., 2015). Conversely, low levels of self-compassion are typically associated with difficulties in emotion regulation and adverse emotional states such as distress, fear of failure, rumination, avoidance, depression, and (social) anxiety (e.g., Adams et al., 2022; Ferrari et al., 2019; MacBeth and Gumley, 2012; Muris and Otgaar, 2023; Tobin and Dunkley, 2021). In contrast, these and similar adverse emotional states often correlate with high levels of self-criticism (Dunkley et al., 2020; Egan et al., 2022; Löw et al., 2020; With et al., 2024).

Psychological problems associated with self-criticism, such as perfectionism and social anxiety, come at a substantial social and psychological cost. For example, perfectionism is associated with higher mortality and suicidal ideation (Fry and Debats, 2009; Smith et al., 2017). Moreover, it has been repeatedly linked to various psychological (e.g., depression, anxiety, and eating disorder; Callaghan et al., 2024; Lunn et al., 2023; Stackpole et al., 2023) and personality disorders (e.g., narcissism, obsessive-compulsive personality disorder; Ayearst et al., 2012; Callaghan et al., 2024; Casale et al., 2024; Dimaggio et al., 2018). Similarly, social anxiety is highly comorbid with psychological disorders such as other anxiety disorders, depression, and substance abuse (Wolitzky-Taylor and LeBeau, 2023).

People suffering from excessive self-criticism, perfectionism, and social anxiety often refrain from seeking adequate treatment (Shannon et al., 2018; Wittchen et al., 1999). Even if treatment is sought, disorders associated with self-criticism are particularly difficult to treat because self-criticism negatively affects the therapeutic alliance (Löw et al., 2020). Although research demonstrates that cognitive-behavioural interventions targeting perfectionism yield moderate to large effect sizes (Callaghan et al., 2024; Galloway et al., 2022; Suh et al., 2019), and various psychological treatments show efficacy for social anxiety disorders (e.g., Mayo-Wilson et al., 2014; Wolitzky-Taylor and LeBeau, 2023), systematic examination of alternative approaches remains limited (Shafran et al., 2023; Wolitzky-Taylor and LeBeau, 2023). Given the transdiagnostic impact of self-criticism, developing further accessible, low-threshold preventive interventions that reach individuals before self-criticism becomes pathological therefore represents a critical need (Dams et al., 2017).

Self-compassion training (SCT) offers a promising alternative by directly targeting self-critical processes through the cultivation of self-kindness and emotional balance (Neff, 2023). SCT have been shown to considerably reduce self-criticism (for a review see Wakelin et al., 2021) and demonstrate moderate to large effect sizes across psychological health outcomes (Ferrari et al., 2019; Finlay-Jones, 2023; Han and Kim, 2023; Kirby et al., 2017). These benefits include improved coping, mood regulation, reduced guilt and shame, and decreased fear of evaluation (Arch et al., 2014; Diedrich et al., 2016; Ehret et al., 2018; Ewert et al., 2021; Leary et al., 2007)—processes central to perfectionism and social anxiety.

Compassion, and self-compassion, has been identified as a critical therapeutic ingredient of perfectionism treatment (Egan et al., 2022; Shafran et al., 2023; Shu et al., 2019). However, research examining specific effects of self-compassion interventions on self-criticism, perfectionism, and social anxiety remains limited. Only preliminary research investigated targeted SCT for reducing perfectionism (e.g., Finlay-Jones et al., 2018; James and Rimes, 2018; Woodfin et al., 2021). Yet, several studies found a reduction in social anxiety and related behavior, such as post-event processing or avoidance, after a self-compassion intervention (Arch et al., 2018; Blackie and Kocovski, 2018; Siegel and Kocovski, 2020; Slivjak et al., 2024). Most of these studies delivered training in face-to-face sessions. Yet, people with a sub-clinical symptom load often fear stigmatization and do not seek sufficient treatment (Schnyder et al., 2017), which should result in a reduced probability of these individuals participating in face-to-face training.

A promising way to provide broad and unobtrusive access to training in health promotion and prevention settings are internet-based psychological interventions. Such interventions encompass many advantages in terms of accessibility and efficiency, time-, and location-independency (i.e., easier integration into participants' daily life), drop-out rate, treatment of subthreshold levels of psychological symptoms, and participants' fear of stigmatization (e.g., Andersson and Titov, 2014; Beecham et al., 2019; Carlbring et al., 2018).

To date, research on internet-based SCT is still in its infancy (Han and Kim, 2023). So far, results suggest positive effects with moderate to large effects on multiple outcomes such as self-compassion, mindfulness, self-esteem, depression, anxiety, stress, self-criticism, and perfectionism (Eriksson et al., 2018; Krieger et al., 2016, Krieger et al., 2019; Nadeau et al., 2021; Stevenson et al., 2019). For example, regarding self-criticism, an 8-week online SCT led to lower levels of self-criticism directly after the training and at a 6-months follow-up (Krieger et al., 2019). Results of a 10-week online SCT targeting perfectionism in a small sample of women suggest that participation in the SCT compared to a waitlist control group resulted in reduced self-judgment, shame, and perfectionism (Nadeau et al., 2021). Moreover, university students who participated in the 5-module online program “Intentional Imperfection Program” which included psychoeducation on self-compassion showed reductions in self-oriented and socially-prescribed perfectionism (Visvalingam et al., 2022). Similarly, undergraduates, who participated in a 2-week online SCT reported reduced shame-proneness, irrational beliefs, and symptoms of social anxiety (Cândea and Szentágotai-Tătar, 2018).

As the research presented above indicates, (online) SCT courses improve a range of psychological health outcomes. However, there is only limited evidence whether online SCT selectively counteracts the negative effects of perfectionism and self-criticism outside of clinical or pre-screened contexts and compared to active control treatments (Han and Kim, 2023; Kirby et al., 2017; Slivjak et al., 2024). Because perfectionistic concerns and self-criticism are arguably conceptual opposites of self-compassion, effects should be comparatively strong and specific (cf. Egan et al., 2022; Shafran et al., 2023; Shu et al., 2019). Given that perfectionism and self-criticism are widespread in the general population and often precede or accompany more severe psychological disorders (Callaghan et al., 2024; Curran and Hill, 2017; Löw et al., 2020), SCT may provide a simple yet effective preventive measure.

The objective of the current study was to examine the efficacy of an online SCT for improving self-compassion, self-criticism, perfectionism, and social anxiety as primary outcomes. Heeding the call for more rigorously designed randomized controlled trials (RCTs) in compassion research (Han and Kim, 2023; Kirby, 2017), we compared the effects of a purpose-designed self-compassion training (SCT) to a generic stress-reduction training (SRT) without explicit self-compassion elements and included a 4-week follow-up. To provide easy access, we implemented the training courses as brief, low-threshold, unsupervised, online interventions consisting of six 15-minute sessions spaced over 2 to 4 weeks.

As primary outcomes, this study focused on self-compassion and the three related constructs self-criticism, perfectionism, and social anxiety. Regarding these primary outcomes, we expected a clear pre–post increase for the SCT and superiority over the SRT. The effect should be strongest for self-compassion and present (if weaker) for the three conceptually related constructs of self-criticism, perfectionism, and social anxiety. As most psychological interventions have broad positive effects (e.g., Borgdorf et al., 2025b; Hildebrandt et al., 2017), we also evaluated the effect of the SCT on general psychological health variables including perceived stress, psychological symptoms, and subjective well-being as secondary outcomes. We expected moderate pre–post improvements on these general variables in both training conditions and no superiority of the SCT over the SRT (cf. van Agteren et al., 2021).

## 2 Method

The anonymized data and analysis code are available on the Open Science Framework (Borgdorf et al., 2025a). Informed consent was obtained from all participants before participation. This study was performed in line with the principles of the Declaration of Helsinki and was approved by the Ethics Committee of the Faculty of Behavioural and Cultural Studies at Heidelberg University, Germany.

### 2.1 Power analysis and participants

Depending on details of the specific analysis, precision-based sample size calculations suggested between 149 and 194 participants to achieve a target CI half-width of 0.20 or 0.25 for Cohen's *d* with 99 % assurance (see Cumming, 2013). For compatibility with null-hypothesis significance testing (NHST) conventions, and to compensate for expected drop-out, we set the sample size a priori to *N* = 200, which yields 80 % power for detecting between-group differences of Cohen's *d* = 0.4 at α = 0.05. As discussed in Lakens (2017), equivalence bounds in the main analysis were set symmetrically to *d* = 0.4, which is the average effect size reported in psychological studies (Richard et al., 2003). This means the study had 80 % power at α = 0.05 to either detect a difference of *d* = 0.4 or to conclude that treatments differ by less than this typical value.

Most participants were recruited online, for example using mailing lists, social media, or bulletin boards. The study language was German, and participant recruitment was focused on Germany, Austria, and Switzerland.

### 2.2 Procedure and participant flow

After reading the study information, confirming their age, and giving informed consent, participants received an individualized link to access the study and training materials on the platform SoSci Survey (Leiner, 2018). The study started with the baseline assessment (T1) including demographic and background information as well as the outcome measures. Upon completion of the baseline assessment, participants were randomly assigned to either the SCT or the SRT by the survey platform. Participants had no prior knowledge on the differences between the training courses, with both being advertised to reduce stress and self-criticism and to be of equal session duration. The randomisation algorithm was designed to “sample without replacement” with the condition of equal distribution to both groups. No additional stratification variables or allocation conditions were implemented. Neither participants nor researchers could influence this automated process.

Both training courses consisted of six online training sessions spread out over 2 to 4 weeks. Every 2 or 3 days, participants received a link to the next session by e-mail, with reminders after 1, 2, and 5 days afterwards. Immediately after the final training session, participants received an invitation to the post assessment (T2) and 28 days later to the follow-up assessment (T3). At the end of the study, participants either received course credit or could register for a lottery to win one of five €50 shopping vouchers.

**Fig. 1:**
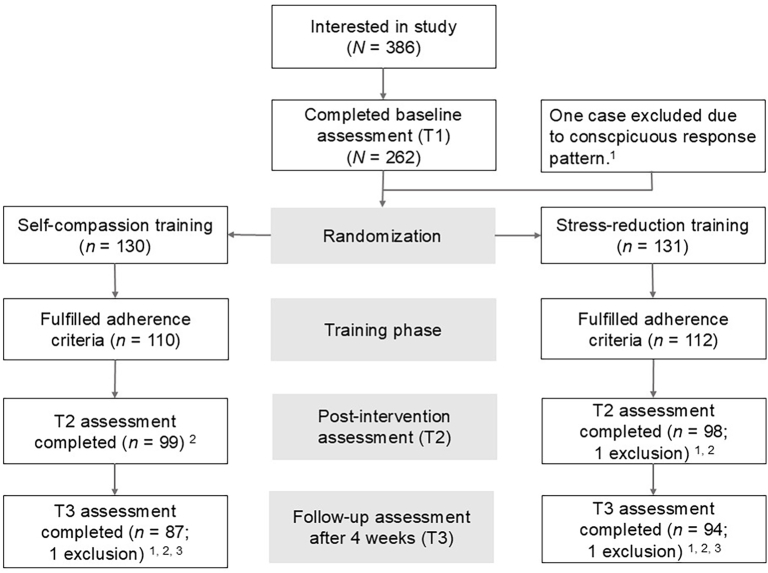
Participant flow. *Note.* A total of *N* = 199 participants were included in the main analyses. ^1^Excluded for exclusive use of a single response category on the Self-Compassion Scale. ^2^Differences in *n* to previous stage: Lost for unknown reasons. ^3^Four participants completed T3 but not T2 and were retained in analyses not requiring data for T2.

Fig. 1 details the structure of the study and the flow of participants according to the CONSORT guidelines (Moher et al., 2010). The drop-out rates between baseline and post-test (SCT: 23.8 %; SRT: 26.0 %) and between baseline and follow-up measurement (SCT: 33.8 %; SRT: 29.0 %) were similar between conditions and comparable to previous online self-compassion intervention studies (e.g., Eriksson et al., 2018; Krieger et al., 2016, Krieger et al., 2019). We included all participants in the analyses who completed at least three out of six training sessions and provided data at baseline (T1) and one or both of the subsequent assessments (T2 or T3), resulting in a final sample size of 200 participants. To investigate the effect of drop-out, we include an intention-to-treat analysis using multiple imputation with all available data from 261 participants in the Supplement.

### 2.3 Training interventions

Participants were randomly assigned either to the purpose-designed SCT or a generic SRT without explicit self-compassion elements. In the first training session (S1), participants watched a short educational video covering key concepts (e.g., “*What is self-compassion?*”, “*How does stress affect the body?*”), and received information on the potential benefits of the upcoming training. The following five training sessions (S2 to S6) provided selected reflections, meditations, guided imagery, or physical exercise tasks in audio, video, or text format. Table 1 depicts an overview and Supplementary Table S1 contains further details of the session content. The purpose of the training sessions was to foster participants' understanding of self-compassion (SRT: mechanisms of stress generation) and provide practical exercises and techniques to enhance self-compassion (SRT: stress management). After each training session, short versions of the exercises were sent to participants by e-mail to encourage self-practice and integration into everyday life.

**Table 1:** Overview of the content of the six training sessions.

	Self-Compassion Training (SCT)	Stress-Reduction Training (SRT)
S1	Introduction video (*What is self-compassion?*) and short reflection task	Introduction video (*How does stress affect the body?*)
S2	Guided meditation (*Self-Compassion Break*, adapted from Neff, 2017)	Written reflection task (stressors and potential ways to reduce them)
S3	Guided meditation (*Affectionate Breathing*, adapted from C. Braehler, 2017a)	Guided imagery (*Boat at the Lake*, adapted from Schellenberg (n.d.-a))
S4	Written reflection task (change of perspective on a negative event, adapted from Leary et al., 2007)	Guided exercise video (yoga-based relaxation exercises by Morrison, 2015)
S5	Guided meditation (*Soften*, *Soothe*, *Allow*; adapted from C. Braehler, 2017b)	Written reflection task (personal values and priorities, adapted from Covey, 2017)
S6	Written reflection task (letter from the compassionate self, adapted from Neff, 2018)	Guided imagery (*Walk at the Beach*, adapted from Schellenberg (n.d.-b))

*Note.* Please refer to Supplementary Table S1 for further details on the training sessions.

Most exercises of the SCT were adapted from audio instructions (e.g., C. Braehler, 2017a, Braehler, 2017b; Neff, 2017, Neff, 2018) based on the Mindful Self-Compassion program by Neff and Germer (2013). The exercises were revised and adapted for the study's purpose and if necessary, translated to German with kind permission of the Center for Mindful Self-Compassion (https://centerformsc.org). More specifically, all SCT exercises directly targeted the three dimensions of self-compassion as defined by Neff (2003): practicing mindfulness of negative experiences, challenging self-critical attitudes with self-kindness, and reflecting on one's own suffering within the context of common humanity. In contrast, the SRT did not include any reference or exercise related to self-kindness, self-criticism, or common humanity. Instead, exercises for the SRT were based on common interventions targeted at reducing stress. These focus on how individuals deal with and perceive stress, using different techniques like modifying behaviours of dealing with stressors (e.g., time management or goal-setting), modifying believes about stress (e.g., cognitive reappraisal; Riepenhausen et al., 2022), modifying the response to stress (e.g., awareness and acceptance of stress; Grossman et al., 2004), or modifying the body's response to stress (e.g., body relaxation techniques; Varvogli and Darviri, 2011). When designing the SRT, we additionally focused on matching the format of the exercises as closely as possible to the SCT.

### 2.4 Measures

All questionnaires were administered in German, required responding on a 5-point Likert scale, and referred to a period of 2 weeks before the assessment, unless stated otherwise. All scales' internal consistencies were good to excellent (Cronbach's α = 0.84 to 0.95; see Supplementary Table S2 for details). Participants filled out all questionnaires before they started the first training session (T1), 2 to 3 days after the final training session (T2), and at a 4-week follow-up (T3).

#### 2.4.1 Self-compassion

Self-compassion was measured with the 26-item Self-Compassion Scale (SCS; Hupfeld and Ruffieux, 2011). The SCS assesses three aspects of self-compassion with two scales each: self-kindness versus self-judgment, common humanity versus isolation, and mindfulness versus over-identification. To simplify analyses, we combined the positive and negative scales for each aspect into three scores.

#### 2.4.2 Self-criticism

The Forms of Self-Criticizing/Attacking and Self-Reassuring Scale (FSCRS; Gilbert et al., 2004) examines how people react when things go wrong for them. The FSCRS is composed of 22 items organized in three scales: Inadequate Self (e.g., “I am easily disappointed with myself.”), Hated Self (e.g.; “I have a sense of disgust with myself.”), and Reassured Self (e.g., “I find it easy to like myself”). We combined these scales into a self-criticism composite with reverse coded Reassured Self items. Carmen Wiencke (Leuphana University Lueneburg, Germany) kindly provided a translated German version because the validated German scale by Biermann et al. (2021) had not been published at the time of data collection.

#### 2.4.3 Perfectionism

Perfectionism was measured with the Concern over Mistakes (COM) and Personal Standards (PS) subscales of the Frost Multidimensional Perfectionism Scale (FMPS; Frost et al., 1990; Altstoetter-Gleich and Bergemann, 2006). COM is reflected by the fear of and sensitivity to making mistakes and failing at one's own standards and consists of eight items (e.g., “If I fail partly, it's as bad as being a complete failure.”). We selected COM as main outcome dimension, as perfectionistic concerns are the typical target of interventions aiming to reduce perfectionism. The PS scale consists of seven items referring to challenging individual goals (e.g., “I set higher goals than most people”). The Self-Oriented Perfectionism (SOPE) subscale of the Hewitt and Flett Multidimensional Perfectionism Scale (HFMPS; Hewitt and Flett, 1991) was also administered, but results are not reported here as it conceptually overlaps with FMPS PS subscale.

#### 2.4.4 Social anxiety

The Social Interaction Anxiety Scale (SIAS; Stangier et al., 1999) measures anxiety in situations of social interaction and is composed of 20 items (e.g., “When mixing socially I am uncomfortable.”).

#### 2.4.5 Psychological health

The Perceived Stress Questionnaire (PSQ; Fliege et al., 2001) measures stress as a subjective feeling without reference to specific events using 20 items with a 4-point Likert scale. For homogeneity of the item format, we reworded items by using the first person (“I” instead of “you”) and changed the present to the past tense (e.g., “I had too many things to do”). Psychological symptom load in the past 7 days was assessed with the Brief Symptom Inventory 18 (BSI-18; Franke et al., 2011), which consists of 18 items covering three symptom clusters (somatization, depression, and anxiety). Finally, the WHO-5 Well-Being Index assesses the presence of positive well-being with five items (WHO, 1998; Braehler et al., 2007). In the analysis, we used overall scores for each of the psychological health measures.

### 2.5 Primary and secondary outcome measures

The measures selected for evaluating the primary outcomes were: Self-Compassion Scale total score, Forms of Self-Criticizing/Attacking & Reassuring Scale total score, Frost Multidimensional Perfectionism Scale COM and PS score, and Social Interaction Anxiety Scale total score. Measures for assessing the secondary outcomes: Perceived Stress Scale, Brief Symptom Inventory total score, and WHO-5 Well-Being Index.

### 2.6 Additional measures

#### 2.6.1 Immediate training effects

To examine the short-term effects of the training, we assessed momentary experiences directly before and after each session (S2 to S5). Participants were asked to indicate on a bipolar scale from −5 to +5 how they felt at that moment in terms of mood (bad – good), energy (tired, weak – awake, full of energy), stress (stressed, tense – relaxed, composed), focus (inattentive, distracted – focused, in the “here and now”), and attitude towards themselves (critical, negative – friendly, positive). The first three items were adapted from the Multi-Dimensional Mood Questionnaire (MDMQ; Wilhelm and Schoebi, 2007).

#### 2.6.2 Satisfaction with the interventions and adverse effects

We examined participants' satisfaction with the training courses using an adapted version of an 8-item scale (ZUF-8) developed by Schmidt et al. (1989; adapted from Krieger et al., 2016). The questionnaire uses 4-point rating scales with anchors adapted to the content of the question (e.g., “How would you generally rate the quality of this training?”). We also asked participants if they experienced any new or aggravated negative effects that they would ascribe to their participation in the study.

### 2.7 Data analyses

Data analyses were performed with the software R version 4.2.2 (R Core Team, 2021; see Supplementary Information for used R packages). The main analysis is based on standardized change scores, representing an unbiased estimate of true change in RCTs (see Jennings and Cribbie, 2021, for a discussion). Following recent recommendations to move beyond null hypothesis significance testing (NHST) for inferential analyses (Wasserstein et al., 2019), we adopted an estimation statistics approach (Cumming, 2013). We suggest interpreting the confidence intervals as uncertainty ranges around effect sizes (see Cumming, 2013), however, they can alternatively be used as tests of significance: A 95 % CI excluding zero corresponds to *p* < 0.05 for a test of difference, a 90 % CI excluding both upper and lower equivalence bounds corresponds to *p* < 0.05 for a test of equivalence (Lakens et al., 2018). Equivalence bounds were set to *d* = [−0.4; 0.4], that is, symmetric to the effect size used for the NHST power calculation mentioned above. Statistical equivalence therefore indicates that the groups differ by less than the typical average effect size of *d* ≈ 0.4 found in most psychological studies (Richard et al., 2003). For compatibility with other statistical approaches, *p*-values and corresponding statistics are reported for the main findings. Regarding false positives due to multiple comparisons, we follow the recommendations of Schulz and Grimes (2005) for handling multiplicity in RCTs through careful interpretation rather than applying potentially inconsistent “correction” procedures.

## 3 Results

The current sample was predominantly female (85.5 %) with a mean age of 30.0 years. All participants indicated that they were proficient German speakers at an advanced level. Table 2 depicts further sociodemographic information. The two training groups did not differ notably on sociodemographic variables or any of the control variables, such as experience with Yoga, Mindfulness-Based Stress Reduction, psychotherapy, counseling, or meditation. The variable with the statistically highest degree of imbalance between conditions was gender, χ^2^(1, *N* = 200) = 2.58, *p* = 0.11.

**Table 2:** Sample characteristics.

	Total (*N* = 200)	SCT (*n* = 100)	SRT (*n* = 100)
Gender (female)	85.5 %	90.0 %	81.0 %
Age (years)^a^			
Mean	30.0	29.2	30.7
Range	18–69	18–64	18–69
Language proficiency (German)			
Native speaker (incl. bilingual)	94.5 %	94.0 %	95.0 %
Secondary language (C1- or C2-level)	5.5 %	6.0 %	5.0 %
Highest education degree			
University	34.0 %	36.0 %	32.0 %
College	56.7 %	57.0 %	56.0 %
Other	9.5 %	7.0 %	12.0 %
Current occupation			
Student	62.0 %	62.0 %	62.0 %
(Self-)Employed	27.0 %	28.0 %	26.0 %
Other	11.0 %	10.0 %	12.0 %
Prior experience			
Psychological therapy or counseling	27.5 %	24.0 %	31.0 %
Mindfulness or relaxation programs^b^	10.5 %	10.0 %	11.0 %
Mindful movement^c^	28.5 %	31.0 %	26.0 %
Meditation experience (10+ h)	24.0 %	24.0 %	24.0 %
Mindfulness practice (at least monthly)	21.5 %	20.0 %	23.0 %

*Note.* SCT = Self-Compassion Training. SRT = Stress-Reduction Training.

a One implausible age value in the SRT was removed.

b e.g., Mindfulness-Based Stress Reduction or Progressive Muscle Relaxation.

c e.g., Yoga or Tai Chi.

### 3.1 Main analyses

Data points exceeding a *z*-score of |3.29| were identified as univariate outliers and winsorized to the nearest non-extreme value (34 out of 12,735 data points). Distribution statistics and inspection of histograms supported the assumption of an approximately normal distribution for all main outcome variables (skewness between −0.70 and 1.10). The online survey platform ensured that there were no missing values in completed questionnaires. Descriptive statistics of all outcomes are depicted in Supplementary Table S3.

**Fig. 2:**
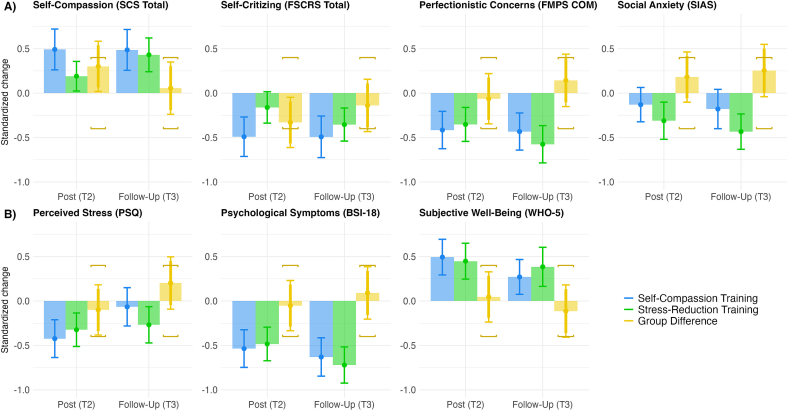
Standardized change scores at post and follow-up measurement by training condition. *Note.* Primary outcomes shown in Panel A), secondary outcomes in Panel B). Solid bars depict standardized change (Cohen's *d*z) relative to T1 for the two conditions (SCT: blue, STR: green) and the difference in standardized change between the two conditions (yellow). Error bars indicate 95 % CIs, the wide segment of error bars additionally show 90 % CIs for group differences. Yellow brackets mark group difference equivalence bounds at Δ*d*z = |0.4|. (For interpretation of the references to color in this figure legend, the reader is referred to the web version of this article.)

The main results are summarized in Fig. 2, which depicts standardized change scores (Cohen's *dz*) with confidence intervals. Precise numbers for statistical test results are reported in Table 3 and Table 4. The general pattern of pre-post comparisons (blue and green bars in Fig. 2) shows statistically relevant favourable changes in both training conditions on nearly all outcome variables. The median absolute effect size across all outcomes and measurement occasions was |*dz*| = 0.39 with an interquartile range (IQR) from 0.27 to 0.49. Notable exceptions were social anxiety and perceived stress at follow-up (T3), showing no discernible effect for the SCT.

**Table 3:** Change scores and statistical test results for baseline (T1) vs. post (T2) comparisons.

Variables	Self-compassion training				Stress-reduction training				Group difference			Equivalence^c^	
	*M* (*SD*)	*dz*^a^	*t*^b^	*p*	*M* (*SD*)	*dz*^a^	*t*^b^	*p*	Δ*dz*	*t*^b^	*p*	*t*^b^	*p*
SCS Total	0.23 (0.55)	0.49	4.25	<0.001	0.09 (0.39)	0.19	2.27	0.03	0.30	2.10	0.04	−0.69	0.25
SCS Self-Kindness	0.28 (0.62)	0.51	4.53	<0.001	0.09 (0.46)	0.17	2.01	0.05	0.34	2.38	0.02	−0.41	0.34
SCS Common Humanity	0.24 (0.66)	0.40	3.63	<0.001	0.07 (0.55)	0.12	1.30	0.20	0.28	1.94	0.05	−0.86	0.20
SCS Mindfulness	0.17 (0.58)	0.33	2.85	<0.01	0.10 (0.41)	0.20	2.38	0.02	0.13	0.93	0.35	−1.86	0.03
FSCRS Total	−0.21 (0.48)	−0.49	−4.38	<0.001	−0.07 (0.38)	−0.16	−1.81	0.07	−0.33	−2.30	0.02	0.49	0.31
FMPS PS	−0.22 (0.50)	−0.47	−4.47	<0.001	−0.17 (0.46)	−0.35	−3.61	<0.001	−0.11	−0.80	0.42	1.99	0.02
FMPS COM	−0.28 (0.70)	−0.41	−3.92	<0.001	−0.24 (0.63)	−0.35	−3.65	<0.001	−0.06	−0.44	0.66	2.35	<0.01
SIAS Total	−0.05 (0.36)	−0.13	−1.33	0.19	−0.11 (0.38)	−0.31	−2.95	<0.01	0.18	1.26	0.21	−1.53	0.06
PSQ Total	−0.17 (0.43)	−0.42	−3.96	<0.001	−0.13 (0.38)	−0.32	−3.41	<0.001	−0.10	−0.70	0.48	2.09	0.02
BSI Total	−0.19 (0.37)	−0.54	−5.02	<0.001	−0.17 (0.33)	−0.48	−5.07	<0.001	−0.05	−0.36	0.72	2.43	<0.01
WHO-5 Total	0.37 (0.75)	0.49	4.88	<0.001	0.33 (0.74)	0.45	4.41	<0.001	0.05	0.32	0.75	−2.48	<0.01

*Note.* SCS = Self-Compassion Scale. PS = Personal Standards Subscale of the Frost Multidimensional Perfectionism Scale (FMPS). COM = Concern over Mistakes Subscale of the FMPS. FSCRS = Forms of Self-Criticizing/Attacking and Self-Reassuring Scale. SIAS = Social Interaction Anxiety Scale. PSQ = Perceived Stress Questionnaire. BSI = Brief Symptom Inventory 18. WHO-5 = WHO-5 Well-Being Index. Means of raw change scores with standard deviations in parentheses. Positive change scores indicate increases from T1 to T2.

a For comparability across groups, the pooled standard deviation of change scores was used for calculating *dz*.

b df_SCT_ = 98, df_STR_ = 96, df_Diff/Equ_ = 194.

c Equivalence bounds at Δ_*dz*_ = 0.4 and −0.4.

**Table 4:** Change scores and statistical test results for baseline (T1) vs. follow-up (T3) comparisons.

Variables	Self-compassion training				Stress-reduction training				Group difference			Equivalence^c^	
	*M* (*SD*)	*dz*^a^	*t*^b^	*p*	*M* (*SD*)	*dz*^a^	*t*^b^	*p*	Δ*dz*	*t*^b^	*p*	*t*^b^	*p*
SCS Total	0.23 (0.52)	0.49	4.21	<0.001	0.21 (0.45)	0.43	4.50	<0.001	0.06	0.37	0.71	−2.31	0.01
SCS Self-Kindness	0.30 (0.60)	0.53	4.61	<0.001	0.21 (0.52)	0.38	3.95	<0.001	0.15	1.00	0.32	−1.68	0.05
SCS Common Humanity	0.20 (0.68)	0.33	2.81	<0.01	0.21 (0.58)	0.33	3.49	<0.001	−0.01	−0.04	0.97	2.64	<0.01
SCS Mindfulness	0.20 (0.58)	0.38	3.26	<0.01	0.20 (0.48)	0.38	4.03	<0.001	0.01	0.06	0.95	−2.62	<0.01
FSCRS Total	−0.24 (0.53)	−0.49	−4.18	<0.001	−0.17 (0.44)	−0.35	−3.77	<0.001	−0.14	−0.93	0.36	1.75	0.04
FMPS PS	−0.34 (0.57)	−0.59	−5.57	<0.001	−0.36 (0.59)	−0.62	−5.92	<0.001	0.03	0.23	0.82	−2.45	<0.01
FMPS COM	−0.31 (0.70)	−0.43	−4.11	<0.001	−0.41 (0.73)	−0.58	−5.45	<0.001	0.14	0.96	0.34	−1.72	0.04
SIAS Total	−0.08 (0.44)	−0.18	−1.61	0.11	−0.18 (0.41)	−0.43	−4.32	<0.001	0.25	1.70	0.09	−0.98	0.16
PSQ Total	−0.03 (0.52)	−0.07	−0.61	0.55	−0.14 (0.51)	−0.27	−2.61	0.01	0.20	1.35	0.18	−1.33	0.09
BSI Total	−0.27 (0.44)	−0.63	−5.80	<0.001	−0.31 (0.43)	−0.72	−7.03	<0.001	0.09	0.61	0.54	−2.07	0.02
WHO-5 Total	0.24 (0.80)	0.27	2.74	<0.01	0.34 (0.94)	0.38	3.46	<0.001	−0.11	−0.76	0.45	1.92	0.03

*Note.* SCS = Self-Compassion Scale. PS = Personal Standards Subscale of the Frost Multidimensional Perfectionism Scale (FMPS). COM = Concern over Mistakes Subscale of the FMPS. FSCRS = Forms of Self-Criticizing/Attacking and Self-Reassuring Scale. SIAS = Social Interaction Anxiety Scale. PSQ = Perceived Stress Questionnaire. BSI = Brief Symptom Inventory 18. WHO-5 = WHO-5 Well-Being Index. Means of raw change scores with standard deviations in parentheses. Positive change scores indicate increases from T1 to T2.

a For comparability across groups, the pooled standard deviation of change scores was used for calculating *dz*.

b df_SCT_ = 86, df_STR_ = 93, df_Diff/Equ_ = 179.

c Equivalence bounds at Δ_*dz*_ = 0.4 and −0.4.

#### 3.1.1 Primary outcomes

The analysis shows a robust pre-post effect of the SCT with respect to self-compassion and self-criticism (Fig. 2, Panel A, blue bars). However, compared to the SRT (Fig. 2, Panel A, yellow bars) the effect was substantially different only directly after the training (T2) but within equivalence bounds at follow-up (T3). Superiority of the SCT regarding self-compassion and self-criticism was therefore at best partially confirmed. The pattern was similar for the related constructs of perfectionistic concerns and social anxiety (Fig. 2, Panel A), but there were no statistically notable differences between groups at any time. Results for perfectionistic strivings (FMPS PS scale) were comparable, see Table 3, Table 4. For social anxiety results were slightly surprising, as pre-post effects of the SCT were nearly zero and the SRT was approaching superiority at follow-up (T3). The intention-to-treat analysis showed the same general pattern (see Supplemental Fig. S5, and Supplemental Tables S6–S7).

#### 3.1.2 Secondary outcomes

Findings for psychological symptoms and subjective well-being (Fig. 2, Panel B) mirror the general pattern evident in the primary outcomes. Both training conditions show clear pre-post effects but are statistically equivalent to each other, which met our expectations regarding these broader outcomes. One exception to this was perceived stress (Fig. 2, Panel B), where the SCT showed no lasting effects at follow-up (T3). The intention-to-treat analysis showed the same general pattern (see Supplemental Fig. S5, and Supplemental Tables S6–S7).

### 3.2 Additional analyses

#### 3.2.1 Immediate training effects

Both interventions produced considerable within-session effects on all state variables, with the difference of pre- and post-session ratings ranging between Cohen's *dz* 0.51 and 1.04 (see Supplementary Table S4 for details). Surprisingly, no meaningful difference between training conditions emerged regarding kind vs. critical attitude towards the self (*diff*_*dz*_ = 0.18, 95 % CI [−0.10, 0.45]).

#### 3.2.2 Participant satisfaction and adverse effects

Overall, participants were similarly satisfied with the SCT (*M* = 3.03, *SD* = 0.54) and the SRT (*M* = 2.93, *SD* = 0.59) at follow-up (T3): *t*(194) = 1.26, *p* = 0.21. Both satisfaction scores were close to the anchor *mostly satisfied* (3 out of 4) of the scale.

Fourteen participants (seven in each condition) reported that they experienced new adverse symptoms during the training period but only two of the participants ascribed the new symptoms to the training without further specifying their complaints. Likewise, 16 participants experienced an aggravation of their symptoms. Of these, four participants (all in the SRT) indicated that this was due to their participation in the study. Two participants reported lower mood or nervousness, the other two criticized the content of the exercises.

### 3.3 Exploratory analyses

#### 3.3.1 Effect of initial self-criticism levels

To investigate whether participants with high initial levels of self-criticism responded particularly well to the SCT, we repeated the main analysis for participants with high “Inadequate Self” (IS) scores on the Forms of Self-Criticizing/Attacking and Self-Reassuring Scale (Gilbert et al., 2004) before the intervention, using the criterion of Krieger et al. (2019; FSCRS IS score ≥ 20). This subsample of 86 participants showed notably stronger and enduring differences between SCT and SRT in self-compassion (T2: Δ_dz_ = 0.60, 95 % CI [0.16, 1.03]; T3: Δ_dz_ = 0.46, 95 % CI [0.00, 0.92]) and self-criticism (T2: Δ_dz_ = −0.71, 95 % CI [−1 0.06, −0.19]; T3: Δ_dz_ = −0.72, 95 % CI [−1.20, −0.27]). However, the effect of selection on group differences in perfectionism, social anxiety, and secondary outcome variables was statistically negligible.

## 4 Discussion

The present randomized controlled study examined the specificity of a brief, low-threshold, unsupervised online self-compassion training (SCT) compared to a matched generic stress-reduction training (SRT). As expected, participation in the SCT led to increases in self-reported self-compassion and a reduction in self-criticism and perfectionism at post-training and 4-week follow-up assessments. Contrary to our expectations, the lasting effect of the SCT on the primary outcomes was not treatment specific. The SCT was marginally superior to the SRT immediately after the intervention regarding self-compassion and self-criticism, but this difference disappeared at 4-week follow-up. For perfectionism and social anxiety there was no statistically meaningful difference between treatments at any time. Moreover, the current findings indicate that both online training courses were effective in maintaining and enhancing subjective well-being and mental health—immediately and up to 4 weeks. Results indicate that participation in all six training sessions of both, SCT and SRT, led to immediate improvements in mood and a more positive self-perception. Additionally, both SCT and SRT had broad positive effects on psychological symptom load, subjective well-being, and—except for the SCT at follow-up—perceived stress. Last, participants in both training conditions were equally satisfied with the two training courses and only a few participants reported minor negative side effects.

We assumed that the SCT would have a specific effect not only on self-compassion but on conceptually related variables when compared to a generic stress-reduction training. Yet, although the SRT did not include any exercises targeting self-critical beliefs or perfectionistic concerns, it led to similar improvements on these variables. Considering this and previous results from research on broad stress-reduction interventions (e.g., Domes et al., 2019; Richardson and Rothstein, 2008), we argue that simply directing attention towards oneself and taking time to consciously unwind from stressful moments may already support the regulation of self-critical thoughts. Spending 20 to 30 min on self-reflection tasks, guided imagery and/or meditation per week may be a crucial factor in preserving and promoting mental well-being (cf. van Agteren et al., 2021). Thus essentially, both training courses were different but similarly effective means to the same end.

Surprisingly, and contrary to previous research (e.g., Krieger et al., 2019), the effects of the SCT on the secondary outcome perceived stress were not maintained at the 4-week follow-up whereas the effects after participation in the SRT were maintained. In contrast, effects on the other secondary outcomes, psychological symptoms and subjective well-being, were stable. A potential explanation may come from the context of self-compassion as an emotion-regulation strategy (e.g., Finlay-Jones, 2023): Self-compassion may not necessarily lead to a reduction in the *perception* of stress but rather to an enhanced effectiveness in *dealing* with it. This explanation is consistent with a study on Mindfulness-Based Cognitive Therapy (MBCT; Kuyken et al., 2010) which shows that increases in self-compassion neutralized the relationship between cognitive reactivity and depression. The study's authors argued that it was not the dysfunctional thinking style per se that was changed but that the response to those thoughts changed through the intervention. In future studies, researchers may hence benefit by focusing on how individuals *deal* with rather than measuring how they *perceive* stress. Consequently, future research should aim to define and differentiate specific and broad effects more systematically, clearly distinguishing between training-specific outcomes and broader, potentially inconsistent psychological changes (Borgdorf et al., 2025b; Hildebrandt et al., 2017; Krieger et al., 2019).

The question of specificity of measures extends to the other outcomes of this study. For example, it could be worthwhile to investigate whether even more specific components, such as cognitive (e.g., perfectionistic cognitions; Flett et al., 1998) or behavioural aspects of perfectionism (e.g., perfectionistic self-presentation tendencies; Hewitt et al., 2003) are better targeted by the SCT than the generic SRT. Also, cognitive components of social anxiety, such as critical post-event processing, may be especially prone to change through an SCT (Blackie and Kocovski, 2018), given that social anxiety in total was not affected by the SCT.

Overall, our finding that neither training was superior to the other is consistent with work on mindfulness-based interventions (MBIs; Mander et al., 2018; Shallcross et al., 2015) and recent meta-analytic results of SCT (Han and Kim, 2023). Studies suggest that MBIs and other SCT are as effective as but not more effective than active control conditions (Palmer et al., 2023). At the same time, our results are in line with research showing that online and mobile-based self-compassion interventions may be as effective as other well-established offline practices, such as cognitive restructuring (Stevenson et al., 2019), mindfulness-based training, or cognitive behavioural psychoeducation (Mak et al., 2018).

Given this pattern of mostly equivalent rather than superior effects, one might question the value of developing additional self-compassion interventions. In terms of cost-benefit considerations, we could arguably focus investment on improving interventions that already exist (Beecham et al., 2019; Shafran et al., 2023). However, when systematically comparing the current findings with established CBT standards for perfectionism treatment, several considerations emerge that may support continued SCT development. Intensive CBT for perfectionism demonstrates medium to large effect sizes (Hedges *g* = 0.57 to 0.89) on perfectionism measures in clinical and subclinical samples. These interventions typically require sustained commitment. On average, they last 7 weeks and include 8 modules (Galloway et al., 2022) of about 50 min to 2 h (Suh et al., 2019). In contrast, the brief, unguided SCT (and SRT) achieved meaningful small to medium reductions on perfectionism (*d* = −0.41 to −0.59) with minimal resources and time investment. Notably, these effects were observed in just 6 sessions of 10–15 min over 2–4 weeks in a non-clinical, non-pre-selected sample.

Although the current SCT demonstrated smaller effect sizes than intensive CBT, this comparison reveals important treatment implications. First, the SCT could serve as an efficient first-step intervention in stepped care models, providing effective and low resource-intensive treatment to individuals before stepping up to more intensive treatments when necessary (Richards et al., 2012). Second, self-compassion interventions may serve as viable alternatives to CBT in highly self-critical samples (cf. Krieger et al., 2019). This is important, because different people may have different preferences and benefit from different approaches. Future research would therefore benefit from comparing the current SCT to established online CBT to determine whether the SCT is comparably effective under similar conditions. Such a comparison would also inform future optimal, evidence-based intervention selection. Additionally, future research employing person-centred approaches to investigating the core mechanisms of interventions, their optimal dose, and combination (or repetition) of exercises could help identify more precisely which treatment works best, for whom, and under which conditions (Blanck et al., 2018; Cha et al., 2022; Goldberg and Davidson, 2024; Goldberg et al., 2020; Palmer et al., 2023). This would ultimately lead to improvements in targeted mental health promotion as well as preventive care (Zilcha-Mano, 2025).

### 4.1 Limitations and future research

The sample investigated in this study consisted of mostly healthy young adults, who are a relevant population for this type of intervention because perfectionism and strong self-criticism have been repeatedly identified as a key mental health issues in this group (Curran and Hill, 2017). Moreover, internet access and experience with web-based applications is very common in this population, which makes the online format particularly feasible. However, the composition of our sample of mostly young, well-educated women, limits generalizability. The current study is also limited by the rather short the follow-up period of 4 weeks, therefore we cannot make assumptions on the long-term stability of the training effects. A similar problem of psychological studies is the use of psychological self-report measures. Although the measures used in this study were reliable and empirically validated indicators, future research could compare other- and self-reports or investigate psychophysiological measures as alternative measures of stress (Di Bello et al., 2020) to compensate for social desirability effects. As mentioned above, more specific investigation of other cognitive, emotional, or behavioural indicators of the target outcomes is warranted.

Finally, we cannot conclude for whom and in which dose either the SCT or the SRT is a better fit. Filtering our sample by initial levels of self-criticism supports the findings of previous studies that people with high levels of self-criticism respond particularly well to self-compassion interventions (e.g., Kelly et al., 2010; Krieger et al., 2019; Stevenson et al., 2019). However, this effect seems to be confined to self-compassion and self-criticism and did not generalize to other outcome variables in our study. Future research may take further individual differences and pre-training (baseline) conditions into account.

## 5 Conclusion

In summary, teaching people to compassionately accept themselves and their imperfections led to improvements in self-compassion, and psychological well-being, as well as considerable decreases in psychological symptom load, self-criticism, and perfectionism. Contrary to former opinions that perfectionism and self-criticism are particularly hard to treat (e.g., Ayearst et al., 2012), our results show that even two brief, low-threshold, unsupervised online training courses can lead to meaningful changes on these variables. Nonetheless, the specific effects and mechanisms of SCT are yet to be identified and understood for the purpose of developing more targeted interventions.

The two brief online training courses may be integrated into more intensive face-to-face programs or therapies, to support and maybe even enhance the effectiveness of these interventions. However, ever since the outbreak of the COVID-19 pandemic, online interventions have become even more relevant. The low-threshold format of our two training courses could easily be scaled up at low costs for mental health promotion and stepped care purposes. For example, the training courses allow for a very feasible and time-efficient integration into various settings, for example in workplace, educational, or health care contexts. Consequently, personal suffering, as well as societal, and economic costs could be reduced.

## CRediT authorship contribution statement

All authors: Conceptualization. [KB]: Methodology, Investigation, Software, Data curation and preparation, Formal Analysis, Writing-Original draft preparation. [CAR]: Supervision, Writing-Reviewing and Editing; [DH]: Formal Analysis, Supervision, Writing-Reviewing and Editing.

## Informed consent

Informed consent was obtained from all individual participants prior to completing the online questionnaires by selecting a mandatory consent checkbox.

## Ethics approval

This study was performed in line with the principles of the Declaration of Helsinki and was approved by the Ethics Committee of the Faculty of Behavioural and Cultural Studies at Heidelberg University, Germany.

## Declaration of Generative AI and AI-assisted technologies in the writing process

During the preparation of this work, the authors used Claude.ai in order to improve language and readability. After using this tool, the authors reviewed and edited the content as needed and take full responsibility for the content of the publication.

## Funding

This research did not receive any specific grant from funding agencies in the public, commercial, or not-for-profit sectors.

## Declaration of competing interest

The authors declare that they have no known competing financial interests or personal relationships that could have appeared to influence the work reported in this paper.

## Data Availability

The anonymized data and analysis script are openly available on the Open Science Framework (OSF) at: https://osf.io/wrhgy/?view_only=f2c6162239324cf889f430ebe1db05ae.

## Appendix A Supplementary data

Supplementary material Image 1
