# Supplementary material for: Effects of a brief online self-compassion training on perfectionism, self-criticism, and social anxiety: A randomized controlled trial
Source: Internet Interv. 2025 Sep 13;42:100870. doi: 10.1016/j.invent.2025.100870 (PMC12862144; doi:10.1016/j.invent.2025.100870)
Supplement: Supplementary file 1 — Supplementary material [file mmc1.pdf]

Supplement

Effects of a brief online self-compassion training on perfectionism, self-criticism, and social anxiety: A randomized controlled trial

Kira S. A. Borgdorf, Corina Aguilar-Raab, & Daniel V. Holt

Table S1

Detailed Overview of the Content of the Six Training Sessions

Session	Self-compassion training	Stress-reduction training
S1	**Educational video:** *What is self-compassion?* https://www.youtube.com/watch?v=2Ho7Svm6ny0 Introduction to the concept of self-compassion, current state of research, its potential benefits, and difference to other self-concepts. **Reflection task**: After the video, participants were asked to reflect on three questions in written form: “What am I suffering from?”; “Are there any negative thoughts or feelings that make the situation even worse?”, and “Why and how could self-compassion be of help for me?”	**Educational video:** *Consequences of stress in the body* https://www.youtube.com/watch?v=Fqnrk6NbB6w Introduction to the concept of stress, its evolutionary development, its possible antecedents and short- and long-term consequences.
S2	**Guided meditation:** *Self-Compassion Break* Participants were instructed to think of the negative or difficult event/situation they reflected on in I1. Having this event in mind, they were guided to reflect on and say three sentences according to the three aspects of self-compassion to themselves (e.g., “This is a difficult moment”; “Suffering is a part of being human”; “May I be friendly to myself”).	**Reflection task:** *Stressors and potential ways to reduce them* Participants were instructed to reflect on stress in their life and write their thoughts down. Firstly, they reflected on stressors in their life. They then were instructed to think of certain life areas (e.g., health, family, social life, etc.) that are affected by this stress. Finally, they thought of possible actions to change or dismiss stressors from their life, or at least, compensate for its consequences.
S3	**Guided meditation:** *Affectionate Breathing* Participants were instructed to be mindful of their breath, to feel the associated movements in the body and to recognize how the breath naturally occurs without any conscious effort. They were guided to be open to and curious on whatever thoughts and feelings may arise during the exercise, to meet those with friendliness and warmth, and to appreciate the natural process of breathing that keeps everyone alive. Whenever their mind wandered, participants were reminded to bring their attention back to their breath in a friendly and understanding way.	**Guided imagery**: *Boat at the Lake* Participants were guided to completely relax and dive into their fantasy world. They received instruction to imagine themselves to be on a wide meadow close to a lake. They were asked to imagine every detail (e.g. what they see, hear, smell, feel) on the meadow, and then follow a path to a boat at a lake. They were asked to enter the boat and take a ride on the lake and feel all their senses in every detail. Afterwards they were instructed to go back to the meadow and come back to the “real world”.

Table S1 (continued)

Detailed Overview of the Content of the Six Training Sessions

Session	Self-compassion training	Stress-reduction training
S4	**Reflection task:** *Change of perspective on a negative event*<br>Participants were asked to reflect on a negative and/or painful event and write their thoughts down. First, they described the event in detail. Participants were then instructed to reflect on their current emotions on this event in an objective manner (SC-mindfulness) and in a next step how other people could experience similar or the same event (common humanity). Finally, they were asked to express and develop a friendly, warm, and understanding inner attitude toward themselves and write some sentences to themselves from this stance (self-kindness).	**Guided exercise video:** *Relaxation exercises*:<br>https://www.youtube.com/watch?v=9gjYZ-2WGOk<br>Participants followed the instructions to perform various physical exercises on a solid chair. The exercises were designed to relief tension in the neck, shoulders, arms, head, and hips. Before starting the exercises, participants were told that they should only follow the exercises that they felt comfortable with and should not push themselves into postures with which they could potentially hurt themselves. In this case they were told to just wait for the next exercise.
S5	**Guided meditation:** *Soften, soothe, allow*<br>In this meditation, participants were instructed to feel their pain caused by negative emotions and thoughts in their body. They named the emotion and explored the feeling in the body. In three steps, participants were then instructed to soften this part of the body, soothe themselves and then allow the negative feeling or discomfort as best as possible, to just "be there" without judging or fighting it.	**Reflection task:** *Values and priorities*<br>Participants were instructed to imagine to be a very old, best possible version of their self who looks back on their life. Participants were asked to reflect on the following questions and write down their answers: "What kind of person have you become being your best possible self?", "What did you achieve in your life and what is important to be remembered?", and "What does this say about your life? What is important to you? Which values can you derive from your reflections?"
S6	**Reflection task:** *Letter from the compassionate self*<br>Participants were instructed to think of a negative and/or painful event which would not overwhelm them. In a first step, participants were asked to think of a close other that experienced the same event and how they would react to that person. After reflecting on this for a few moments, participants were asked to transfer those feelings of understanding, warmth and caring to themselves and write themselves a compassionate letter from this caring and friendly stance.	**Guided imagery:** *Walk at the Beach*<br>Participants were guided to completely relax and dive into their fantasy world (cf. I3). They were guided to first sit in the sand and then take a walk at the beach and meanwhile feel all their senses.

Table S2

Scale Reliabilities (Cronbach's α)

Measure	T1 (*N* = 200)	T2 (*N* = 196)	T3 (*N* = 181)
SCS Total	0.94	0.95	0.94
SCS Self-Kindness	0.91	0.92	0.92
SCS Common Humanity	0.81	0.83	0.84
SCS Mindfulness	0.83	0.85	0.83
FSCRS Total	0.92	0.93	0.93
PS	0.84	0.87	0.86
COM	0.90	0.90	0.90
SIAS Total	0.86	0.88	0.88
PSQ Total	0.90	0.92	0.91
BSI Total	0.87	0.88	0.89
WHO-5 Total	0.84	0.86	0.87

Note. SCS = Self-Compassion Scale. PS = Personal Standards Subscale of the Frost Multidimensional Perfectionism Scale (FMPS). COM = Concern over Mistakes Subscale of the FMPS. FSCRS = Forms of Self-Criticizing/Attacking and Self-Reassuring Scale. SIAS = Social Interaction Anxiety Scale. PSQ = Perceived Stress Questionnaire. BSI = Brief Symptom Inventory 18.WHO-5 = WHO-5 Well-Being Index.

Table S3

Descriptive Statistics for T1, T2 and T3

Variables	Self-Compassion Training			Stress-Reduction Training		
	T_1	T_2	T_3	T_1	T_2	T_3
SCS Total	3.03 (0.75)	3.28 (0.72)	3.30 (0.67)	3.08 (0.70)	3.16 (0.78)	3.31 (0.77)
SCS Self-Kindness	3.01 (0.79)	3.30 (0.80)	3.33 (0.72)	3.05 (0.83)	3.14 (0.87)	3.29 (0.88)
SCS Common Humanity	3.07 (0.83)	3.32 (0.77)	3.30 (0.76)	3.10 (0.74)	3.17 (0.82)	3.33 (0.86)
SCS Mindfulness	3.02 (0.85)	3.22 (0.78)	3.27 (0.72)	3.08 (0.73)	3.17 (0.83)	3.31 (0.79)
FSCRS Total	2.79 (0.83)	2.57 (0.77)	2.50 (0.71)	2.72 (0.74)	2.66 (0.82)	2.54 (0.86)
Perfectionism Composite	3.09 (0.75)	2.88 (0.69)	2.78 (0.73)	3.13 (0.73)	3.01 (0.74)	2.81 (0.74)
PS	3.42 (0.78)	3.17 (0.77)	3.08 (0.80)	3.46 (0.77)	3.33 (0.82)	3.09 (0.88)
COM	2.54 (1.04)	2.26 (0.94)	2.17 (0.99)	2.59 (1.00)	2.36 (0.95)	2.16 (0.88)
SIAS Total	2.42 (0.72)	2.37 (0.72)	2.33 (0.74)	2.49 (0.80)	2.36 (0.78)	2.26 (0.76)
PSQ Total	2.43 (0.60)	2.25 (0.63)	2.40 (0.60)	2.50 (0.62)	2.34 (0.62)	2.35 (0.61)
BSI Total	1.92 (0.58)	1.74 (0.51)	1.63 (0.52)	1.96 (0.60)	1.77 (0.58)	1.65 (0.55)
WHO-5 Total	3.52 (0.90)	3.88 (0.84)	3.77 (0.89)	3.42 (1.03)	3.78 (0.98)	3.74 (1.01)

Note. SCS = Self-Compassion Scale. PS = Personal Standards Subscale of the Frost Multidimensional Perfectionism Scale (FMPS). COM = Concern over Mistakes Subscale of the FMPS. FSCRS = Forms of Self-Criticizing/Attacking and Self-Reassuring Scale. SIAS = Social Interaction Anxiety Scale. PSQ = Perceived Stress Questionnaire. BSI = Brief Symptom Inventory 18.WHO-5 = WHO-5 Well-Being Index. Means of raw scores with standard deviations in parentheses. Sample sizes: $n_{SCT\_T1} = 100$, $n_{SCT\_T2} = 99$, $n_{SCT\_T3} = 87$, $n_{STR\_T1} = 100$, $n_{STR\_T2} = 97$, $n_{STR\_T3} = 94$.

Table S4

Immediate Training Effects

	SCT (n = 100)						SRT (n = 100)						Group Difference			
Variables *M*(*SD*)	S2	S3	S4	S5	S6	$dz_{pre\text{-}post}$ [a]	S2	S3	S4	S5	S6	$dz_{pre\text{-}post}$ [a]	Δdz	t [b]	*95% CI*	p
Mood (pre)	7.86 (2.11)	7.51 (2.25)	7.90 (2.01)	7.94 (2.00)	7.79 (2.02)	0.56	7.60 (2.19)	7.53 (2.41)	7.90 (1.96)	7.73 (2.32)	7.87 (2.25)	0.51	0.05	0.36	[−0.23, 0.33]	.72
Mood (post)	8.25 (1.97)	8.43 (1.94)	7.91 (2.11)	8.32 (1.96)	8.53 (1.93)		7.77 (2.22)	8.40 (2.01)	8.28 (1.94)	8.13 (2.31)	8.16 (2.11)					
Energy (pre)	5.56 (2.5)	5.66 (2.72)	6.68 (2.44)	6.74 (2.53)	7.04 (2.54)	0.72	5.63 (2.49)	5.78 (2.57)	5.89 (2.49)	6.39 (2.45)	6.68 (2.63)	1.04	−0.32	−2.32	[−0.60, −0.05]	.02
Energy (post)	6.3 (2.41)	6.83 (2.45)	7.19 (2.31)	7.16 (2.54)	7.82 (2.24)		6.51 (2.39)	7.00 (2.12)	7.54 (2.30)	7.36 (2.24)	7.22 (2.45)					
Stress (pre)	6.56 (2.56)	6.73 (2.73)	7.17 (2.27)	7.15 (2.54)	7.19 (2.58)	0.79	6.38 (2.68)	6.42 (2.61)	7.17 (2.44)	6.87 (2.41)	7.08 (2.66)	0.88	−0.09	−0.64	[−0.37, 0.19]	.52
Stress (post)	7.71 (2.43)	8.21 (2.36)	7.42 (2.24)	7.89 (2.15)	7.96 (2.12)		7.00 (2.38)	7.70 (2.47)	8.11 (2.24)	7.92 (2.44)	7.94 (2.44)					
Focus (pre)	6.59 (2.38)	6.96 (2.20)	7.29 (2.05)	7.40 (2.15)	7.59 (2.20)	0.64	6.96 (2.60)	6.69 (2.5)	6.99 (2.26)	6.96 (2.6)	7.25 (2.48)	0.82	−0.19	−1.32	[−0.46, 0.09]	.19
Focus (post)	7.73 (2.20)	7.66 (2.09)	7.74 (2.04)	7.85 (2.11)	8.47 (1.83)		7.68 (2.30)	7.53 (2.60)	8.23 (1.77)	8.12 (2.28)	7.80 (2.24)					
Self-Criticism (pre)	7.06 (2.43)	7.51 (2.37)	7.68 (2.03)	7.87 (1.94)	7.63 (2.21)	0.88	7.26 (2.41)	7.63 (2.37)	7.68 (2.12)	7.65 (2.33)	7.69 (2.34)	0.71	0.18	1.25	[−0.10, 0.45]	.21
Self-Criticism (post)	7.89 (1.99)	8.32 (2.06)	8.23 (2.09)	8.28 (1.84)	8.48 (2.00)		7.70 (2.21)	8.29 (2.13)	8.24 (2.00)	8.23 (2.29)	8.22 (2.2)					

Note. SCT = Self-Compassion Training. SRT = Stress-Reduction Training. [a] For comparability across groups, the pooled standard deviation of change scores was used for calculating *dz*. [b] df = 198.

Figure S5

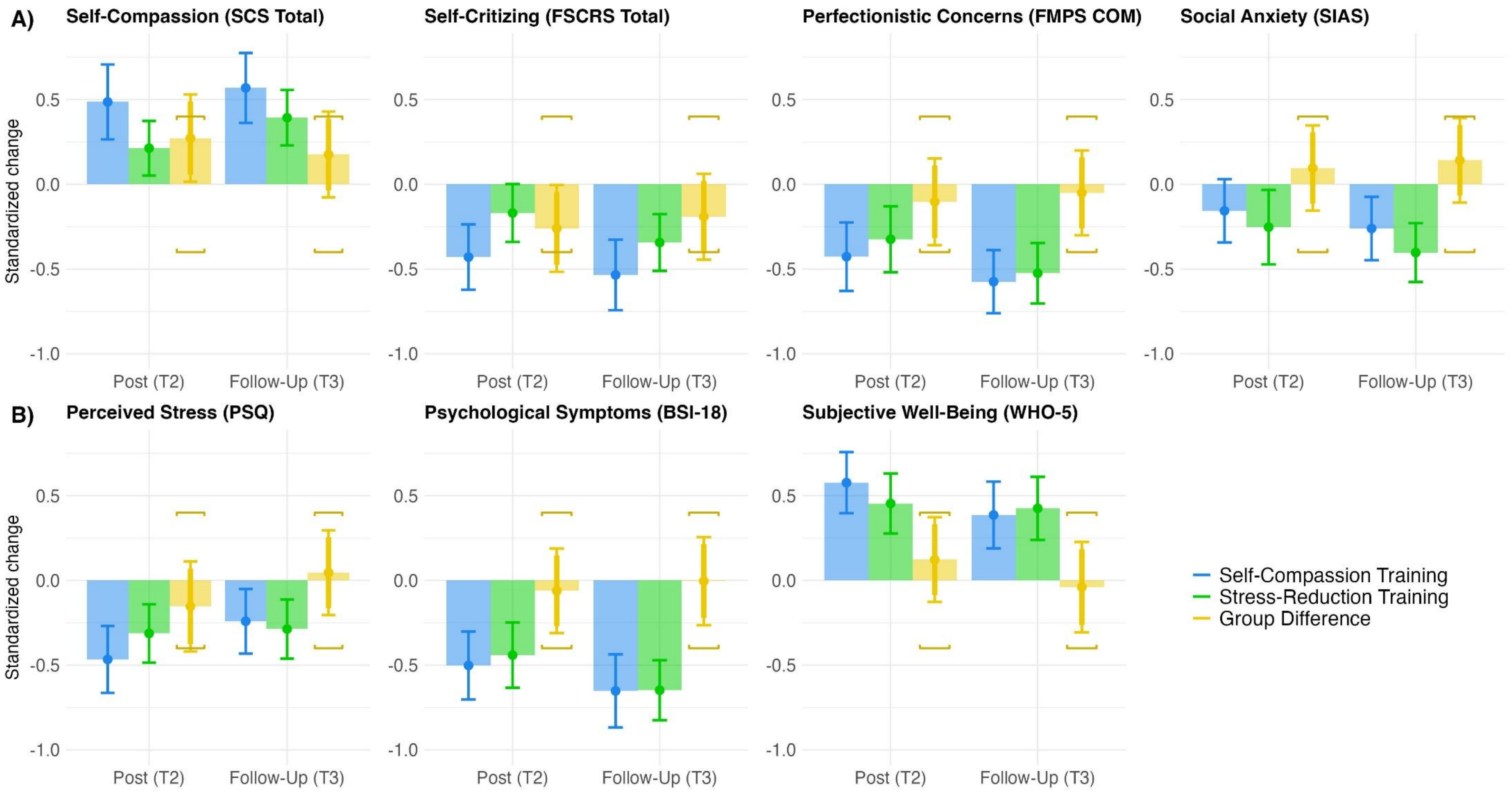

Note. Figure showing main results based on an intention-to-treat analysis including $N = 261$ participants. Missing data were imputed using the R mice package with m = 5 imputations. Primary outcomes shown in Panel A), secondary outcomes in Panel B). Solid bars depict standardized change (Cohen's dz) relative to T1 for the two conditions (SCT: blue, STR: green) and the difference in standardized change between the two conditions (yellow). Error bars indicate 95 % CIs, the wide segment of error bars additionally show 90 % CIs for group differences. Yellow brackets mark group difference equivalence bounds at $\Delta dz = |0.4|$.

Table S6

Change scores and statistical test results for baseline (T1) vs. post (T2) comparisons based on an intention-to-treat analysis including N = 261 participants. Missing data were imputed using the R mice package with m = 5 imputations.

Variables	Self-Compassion Training				Stress-Reduction Training				Group Difference			Equivalence[c]	
	M (*SD*)	*dz*[a]	*t*[b]	*p*	*M* (*SD*)	*dz*[a]	*t*[b]	*p*	Δ*dz*	*t*[b]	*p*	*t*[b]	*p*
SCS Total	0.31 (0.68)	0.49	4.31	<.001	0.13 (0.57)	0.21	2.59	.01	0.27	2.08	.04	2.08	.17
SCS Self−Kindness	0.33 (0.81)	0.43	3.88	<.001	0.12 (0.72)	0.16	1.63	.12	0.26	1.92	.06	1.92	.16
SCS Common Humanity	0.32 (0.81)	0.41	4.28	<.001	0.14 (0.76)	0.18	1.95	.05	0.23	1.73	.09	1.73	.10
SCS Mindfulness	0.27 (0.84)	0.36	2.94	< .01	0.14 (0.63)	0.19	2.45	.02	0.17	1.18	.24	1.18	.06
FSCRS Total	−0.24 (0.62)	−0.43	−4.36	<.001	−0.10 (0.51)	−0.17	−1.94	.06	−0.26	−1.99	.05	−1.99	.14
FMPS PS	−0.32 (0.78)	−0.43	−4.15	<.001	−0.24 (0.71)	−0.32	−3.26	< .01	−0.10	−0.79	.43	−0.79	.01
FMPS COM	−0.21 (0.56)	−0.39	−3.54	< .01	−0.12 (0.51)	−0.23	−2.56	.01	−0.15	−1.03	.31	−1.03	.06
SIAS Total	−0.07 (0.42)	−0.16	−1.64	.11	−0.11 (0.44)	−0.25	−2.26	.03	0.10	0.75	.45	0.75	< .01
PSQ Total	−0.21 (0.49)	−0.47	−4.62	<.001	−0.14 (0.43)	−0.31	−3.56	<.001	−0.15	−1.13	.26	−1.13	.04
BSI Total	−0.20 (0.42)	−0.50	−4.91	<.001	−0.18 (0.39)	−0.44	−4.48	<.001	−0.06	−0.48	.63	−0.48	< .01
WHO−5 Total	0.45 (0.78)	0.58	6.27	<.001	0.35 (0.78)	0.45	5.03	<.001	0.12	0.96	.34	0.96	.02

Note. SCS = Self-Compassion Scale. PS = Personal Standards Subscale of the Frost Multidimensional Perfectionism Scale (FMPS). COM = Concern over Mistakes Subscale of the FMPS. FSCRS = Forms of Self-Criticizing/Attacking and Self-Reassuring Scale. SIAS = Social Interaction Anxiety Scale. PSQ = Perceived Stress Questionnaire. BSI = Brief Symptom Inventory 18.WHO-5 = WHO-5 Well-Being Index. Means of raw change scores with standard deviations in parentheses. Positive change scores indicate increases from T1 to T2. [a] For comparability across groups, the pooled standard deviation of change scores was used for calculating *dz*. [b] dfs vary due to imputation adjustments. [c] Equivalence bounds at $\Delta_{dz} = 0.4$ and $\Delta_{dz} = -0.4$.

Table S7

Change scores and statistical test results for baseline (T1) vs. follow-up (T3) comparisons based on an intention-to-treat analysis including N = 261 participants. Missing data were imputed using the R mice package with m = 5 imputations.

Variables	Self-Compassion Training				Stress-Reduction Training				Group Difference			Equivalence[c]	
	M (*SD*)	*dz*[a]	*t*[b]	*p*	*M* (*SD*)	*dz*[a]	*t*[b]	*p*	Δ*dz*	*t*[b]	*p*	*t*[b]	*p*
SCS Total	0.34 (0.64)	0.57	5.40	<.001	0.24 (0.56)	0.39	4.71	<.001	0.18	1.36	.17	1.36	.04
SCS Self−Kindness	0.40 (0.74)	0.57	5.01	<.001	0.27 (0.66)	0.38	4.12	<.001	0.18	1.41	.16	1.41	.05
SCS Common Humanity	0.31 (0.75)	0.43	4.31	<.001	0.23 (0.69)	0.32	3.62	<.001	0.11	0.81	.42	0.81	.02
SCS Mindfulness	0.31 (0.71)	0.48	4.65	<.001	0.24 (0.57)	0.37	4.56	<.001	0.10	0.81	.42	0.81	.01
FSCRS Total	−0.32 (0.67)	−0.53	−5.04	<.001	−0.21 (0.55)	−0.34	−4.02	<.001	−0.19	−1.48	.14	−1.48	.05
FMPS PS	−0.46 (0.83)	−0.57	−6.04	<.001	−0.42 (0.77)	−0.52	−5.75	<.001	−0.05	−0.39	.69	−0.39	< .01
FMPS COM	−0.38 (0.63)	−0.60	−5.66	<.001	−0.32 (0.63)	−0.51	−5.62	<.001	−0.09	−0.68	.50	−0.68	.01
SIAS Total	−0.13 (0.51)	−0.26	−2.73	< .01	−0.20 (0.47)	−0.40	−4.55	<.001	0.14	1.12	.27	1.12	.02
PSQ Total	−0.14 (0.61)	−0.24	−2.48	.02	−0.17 (0.58)	−0.29	−3.22	< .01	0.05	0.36	.72	0.36	< .01
BSI Total	−0.33 (0.53)	−0.65	−5.92	<.001	−0.33 (0.49)	−0.65	−7.18	<.001	−0.00	−0.03	.98	−0.03	< .01
WHO−5 Total	0.36 (0.89)	0.39	3.84	<.001	0.39 (0.96)	0.43	4.47	<.001	−0.04	−0.29	.77	−0.29	< .01

Note. SCS = Self−Compassion Scale. PS = Personal Standards Subscale of the Frost Multidimensional Perfectionism Scale (FMPS). COM = Concern over Mistakes Subscale of the FMPS. FSCRS = Forms of Self−Criticizing/Attacking and Self−Reassuring Scale. SIAS = Social Interaction Anxiety Scale. PSQ = Perceived Stress Questionnaire. BSI = Brief Symptom Inventory 18.WHO−5 = WHO−5 Well−Being Index. Means of raw change scores with standard deviations in parentheses. Positive change scores indicate increases from T1 to T2. [a] For comparability across groups, the pooled standard deviation of change scores was used for calculating *dz*. [b] dfs vary due to imputation adjustments. [c] Equivalence bounds at $\Delta_{dz} = 0.4$ and $\Delta_{dz} = -0.4$.

R packages

Revelle, W. (2023). *psych: Procedures for psychological, psychometric, and personality research* (Version 2.3.9) [R package]. Northwestern University. https://CRAN.R−project.org/package=psych

Wilke, C. O. (2023). *cowplot: Streamlined plot theme and plot annotations for 'ggplot2'* (Version 1.1.2) [R package]. https://CRAN.R−project.org/package=cowplot

Wickham, H. (2016). *ggplot2: Elegant graphics for data analysis* (2nd ed.). Springer. https://doi.org/10.1007/978−3−319−24277−4

Wickham, H., Averick, M., Bryan, J., Chang, W., McGowan, L. D., François, R., Grolemund, G., Hayes, A., Henry, L., Hester, J., Kuhn, M., Pedersen, T. L., Miller, E., Bache, S. M., Müller, K., Ooms, J., Robinson, D., Seidel, D. P., Spinu, V., ... & Yutani, H. (2019). Welcome to the tidyverse. *Journal of Open Source Software, 4*(43), 1686. https://doi.org/10.21105/joss.01686
